# Genome-wide association study of coleoptile length with Shanxi wheat

**DOI:** 10.3389/fpls.2022.1016551

**Published:** 2022-09-21

**Authors:** Naicui Wei, ShengQuan Zhang, Ye Liu, Jie Wang, Bangbang Wu, Jiajia Zhao, Ling Qiao, Xingwei Zheng, Juanling Wang, Jun Zheng

**Affiliations:** ^1^School of Life Sciences, Shanxi University, Taiyuan, China; ^2^Institute of Hybrid Wheat, Beijing Academy of Agriculture and Forestry Sciences, Beijing, China; ^3^State Key Laboratory of Sustainable Dryland Agriculture, Institute of Wheat Research, Shanxi Agricultural University, Linfen, China

**Keywords:** Shanxi wheat, coleoptile length, drought stress, GWAS, 3VmrMLM

## Abstract

In arid and semi-arid regions, coleoptile length is a vital agronomic trait for wheat breeding. The coleoptile length determines the maximum depth that seeds can be sown, and it is critical for establishment of the crop. Therefore, identifying loci associated with coleoptile length in wheat is essential. In the present study, 282 accessions from Shanxi Province representing wheat breeding for the Loess Plateau were grown under three experimental conditions to study coleoptile length. The results of phenotypic variation indicated that drought stress and light stress could lead to shortening of coleoptile length. Under drought stress the growth rate of environmentally sensitive cultivars decreased more than insensitive cultivars. The broad-sense heritability (*H*^2^) of BLUP (best linear unbiased prediction) under various conditions showed G × E interaction for coleoptile length but was mainly influenced by heredity. Correlation analysis showed that correlation between plant height-related traits and coleoptile length was significant in modern cultivars whereas it was not significant in landraces. A total of 45 significant marker-trait associations (MTAs) for coleoptile length in the three conditions were identified using the 3VmrMLM (3 Variance-component multi-locus random-SNP-effect Mixed Linear Model) and MLM (mixed linear model). In total, nine stable genetic loci were identified via 3VmrMLM under the three conditions, explaining 2.94–7.79% of phenotypic variation. Five loci on chromosome 2B, 3A, 3B, and 5B have not been reported previously. Six loci had additive effects toward increasing coleoptile length, three of which are novel. Molecular markers for the loci with additive effects on coleoptile length can be used to breed cultivars with long coleoptiles.

## Introduction

The coleoptile is a conical sheath-like structure that protects the young cotyledons and apical meristem of monocotyledonous plants during germination and emergence (Pucciariello, 2020). The wheat coleoptile is composed of inner and outer epidermis with vascular bundles and long and narrow cells, mostly parenchyma, covered by stratum corneum (Hohl and Schopfer, 1992; Liao et al., 2011). In arid and semi-arid regions, coleoptile length is a vital agronomic trait in wheat breeding. It is critical for wheat establishment because its length determines the maximum depth that seeds can be sown (Li et al., 2017). Wheat seedling emergence and survival in arid areas is dependent on sufficient soil moisture. Seedlings emergence will be uneven or even fail when the soil moisture content is lower than 16–18% (Li et al., 2014). Especially in years with less rainfall, wheat tends to be sown deeper (Mohan et al., 2013; Francki et al., 2021). Wheat cultivars with long coleoptiles can be sown deep to ensure that the seeds can absorb enough moisture for germination and stand establishment, thereby enabling normal yield (Anzooman et al., 2018). Therefore, dissecting the genetic basis of coleoptile length is important for the improvement of wheat in arid and semi-arid regions.

Differences in moisture and temperature leads to the differences in coleoptile length in various regions. The wheat growing areas of the north central United States have a continental climate, with severe winters, hot summers, and a short rainy season. There is no direct and indirect interference in breeding, so the average length of wheat coleoptiles was about 7 cm (Sidhu et al., 2020). However, the rainfall in southeast and southwest of Australia was evenly distributed, with a long rainy season. Wheat cultivars with long coleoptiles were preferred in breeding, resulting in cultivars with an average coleoptile length of 8–11 cm (Rebetzke et al., 2001, 2014). The coleoptile length of wheat is a quantitative trait under polygenic control. It is influenced by both genotype and environment and can be adjusted by genetic improvement (Yang et al., 2007). With the development of molecular biology and genomics in the field of wheat breeding, some progress had been made in QTL mapping of coleoptile length. Quantitative trait loci (QTL) for traits related to coleoptile length have been reported on all chromosomes by using bi-parental and multi-parental mapping populations. The pleiotropic genes *Rht1* and *Rht2*, located, respectively, on chromosomes 4B and 4D, reduce both coleoptile length and plant height (Rebetzke et al., 2004, 2007a). Using recombinant inbred lines (RILs), main effect QTL related to coleoptile length, such as *qCL.3B.1* and *qCL.4B.1*, were found to account for 6.6–19.9% of the phenotypic variation (Singh et al., 2015). Also using RILs, a major QTL on chromosomes 4DL was found to explain 33% of phenotypic variation, and *Rht1* and *Rht2* were also detected (Yu and Bai, 2010). Genome-wide association study (GWAS) is another widely used approach that can reduce the limitations of bi-parental or multi-parental QTL identification. The dwarf genes *Rht1* and *Rht2* were located via association analysis using a global wheat collection of 893 accessions grown in various environments (Li et al., 2016). Seventeen marker-trait associations (MTAs) for coleoptile length were identified in 707 Chinese landraces, explaining 2.73–8.17% of the phenotypic variation (Ma et al., 2020). A study in the United States using association analysis of a diverse population of 473 wheat cultivars and a population of 279 wheat breeding lines identified three major markers of environmental stability for coleoptile length, which explained 4–7% of the phenotypic variation (Merrick et al., 2021). However, there are few studies reported using GWAS to identify novel loci for coleoptile length. Few of these GWAS literatures address the relationship between coleoptile length and plant height-related agronomic traits. Although association analysis has many advantages, such as being less time-consuming with large allele coverage and high mapping accuracy, the false positive rate of the loci identified is high (Zhang et al., 2006; Yang et al., 2007). In addition, most models of association analysis do not decompose additive and dominant effects, resulting in loci that cannot be stably identified across environments (Wang et al., 2016; Wen et al., 2018). The 3VmrMLM (3 Variance-component multi-locus random-SNP-effect Mixed Linear Model) distinguishes various confounding effects by pre-estimating the genotype effect, and is more conducive to the detection of loci (Li et al., 2022). Only a few studies have used GWAS to identify novel loci for coleoptile length compared with other major agronomic traits such as plant height, thousand-grain weight, and grain number per spike. Furthermore, some of the markers used were SSR, which are few in number and low in density, and there is no report on the application of markers to breeding for coleoptile length (Spielmeyer et al., 2007). Therefore, using more effective methods to identify QTL for coleoptile length is of great significance for wheat improvement.

The Loess Plateau is famous for the world’s largest and deepest loess deposits (Fu et al., 2017). Shanxi Province in China is in the Loess Plateau, accounting for more than a quarter of the Loess Plateau’s total area (Wang et al., 2022). This region has a long history of wheat cultivation, but water resources are scarce. Shanxi province has annual rainfall between 400 and 650 mm, which is typical for semi-arid regions (Zheng et al., 2021). Therefore, Shanxi wheat cultivars are suitable for studying QTL for wheat drought resistance-related agronomic traits. The present study is needed because (1) the number of loci and markers for wheat coleoptile length is low compared to other agronomic traits, (2) the relationship between coleoptile length and plant height-related agronomic traits is unclear, and (3) it is unclear whether light and dark treatments affect coleoptile length differences among genotypes. With a GWAS approach, 241 modern cultivars, 41 landraces, and 3VmrMLM analysis were used to identify significant loci of coleoptile length, and provide a basis for molecular marker-assisted selection for coleoptile length in wheat.

## Materials and methods

### Plant materials

A diverse hexaploid wheat collection of 282 accessions from Shanxi Province was used (Supplementary Table 1). These genotypes have significant phenotypic differences and include 126 irrigated and 115 dryland modern wheat cultivars. The remaining 41 experimental materials were landraces from Shanxi Province selected from the Chinese wheat core collection (Zheng et al., 2021).

### Coleoptile length measurement

Seeds of each line were carefully cleaned with 3% H_2_O_2_ and distilled water and evaluated in three treatments (C_1_–C_3_; Sidhu et al., 2020). C_1_ condition represents normal control with three replicates (E_1_–E_3_), C_2_ condition represents drought treatment with two replicates (E_5_–E_6_), and C_3_ condition represents light treatment with two replicates (E_8_–E_9_). E_4_, E_7_, and E_10_ are the mean values of each treatment, respectively. C_1_:E_1_–E_4_ 20°C in darkness with 150 ml distilled water; C_2_:E_5_–E_7_ 20°C in darkness with 150 ml, 20% PEG-6000 hypertonic solution to simulate drought stress; C_3_:E_8_–E_10_ 20°C, 12 h darkness + 12 h light at 500μmol⋅m^–2^⋅s^–1^ with 150 ml distilled water. In each replicate, twenty seeds per genotype were placed and germinated on a filter paper measuring 40 cm × 60 cm. After seven days, the coleoptile lengths of five individual plants for each cultivar were measured. Coleoptile length was recorded as the distance from the seed to where the first leaf broke through the coleoptile sheath (Bai et al., 2004). For all 282 accessions, data were collected for plant height, uppermost internode length, and internode length of basal sections I–IV from 2020 to 2021 field plots grown in Yaodu District (P_1_:36°48′N, 111°30′E) and Yanhu District (P_2_:35°20′N, 110°59′E) in Shanxi Province.

### Genomic deoxyribonucleic acid extraction and genotyping

Genomic DNA was extracted using the cetyl trimethyl ammonium bromide (CTAB) method (Zheng et al., 2019). DNA was sent to the MOL-BREEDING Company (Shijiazhuang, China) for high-throughput genotyping using wheat GenoBaits technology (14868 mSNP, 37669 SNP). After eliminating markers with an allele frequency (MAF) < 0.05, > 10% missing data, or > 20% heterozygosity (Arora et al., 2017; Jung et al., 2021), a total of 9,793 high-quality SNPs were identified for GWAS analyses (Zheng et al., 2021).

### Genome-wide association analysis

Two models were used for association analysis between coleoptile length and SNP markers. Marker-trait associations were tested using the 3VmrMLM in the RStudio software and markers with a threshold of LOD score ≥3.99 were considered as significant associations (Wang et al., 2016; Li et al., 2022). Similarly, marker-trait associations were tested using the mixed linear model (MLM) in TASSEL 5.0 software and markers with a threshold of -log_10_(*p*-value) ≥ 3.99 were used as the screening criterion (Zhang et al., 2010; Li et al., 2016). GWAS was conducted with ten datasets, including E_1_ to E_10_. Manhattan was generated via RStudio software to visualize the GWAS results. The linkage disequilibrium (LD) of each SNP marker was extended on each chromosome (Zheng et al., 2021). The extended region where the LD between nearby SNPs and the peak SNP decayed to *R*^2^ = 0.2 was defined as the local LD-based QTL interval (Zheng et al., 2019). For each associated locus, the *p*-value and QTL intervals of the peak SNP defined the significance and the interval of the locus (Zhang et al., 2016).

### Gene analysis of functional interval

To facilitate the identification of genes in functional regions, the chromosomal region was delimited at 6.11 Mb (Yao et al., 2021). Genes within the target region were identified using the genome browser (JBrowse) on the WheatOmics-bata website^1^ (Ma et al., 2021). The sequences of common wheat genes were retrieved based on the intervals of major QTL identified from https://urgi.versailles.inra.fr/download/iwgsc/IWGSC_RefSeq_Annotations/v1.0/ (Zheng et al., 2019). Functional annotation of genes in segments were carried out in the Gene Ontology (GO) database using the R package cluster Profiler. The RNA-seq of genes within the target region were retrieved from Wheat Expression Browser.^2^ Statistical calculations of phenotypic data were performed by SAS V8.0^3^ (Smith et al., 1998). BLUP calculation, phenotypic variance analysis, and correlation analysis were performed by JMP Pro16^4^ and IBM SPSS Statistics 26 software.^5^ Data was plotted via RStudio^6^ and Origin 2018.^7^

## Results

### Phenotypic variation of coleoptile length

**FIGURE 1:**
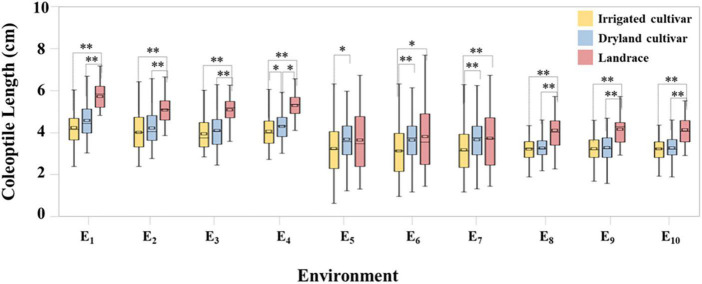
Coleoptile length of irrigated cultivars, dryland cultivars, and landraces. * and ^**^ represent significance level of *P* < 0.05 and *P* < 0.01, respectively.

Coleoptile length within the 282 wheat accessions was normally distributed across in all ten datasets (Supplementary Figure 1). Under the C_1_ condition, coleoptile lengths ranged from 2.80 to 7.03 cm and the coefficient of variation was 17.78%, indicating that there were significant differences in coleoptile length among the test materials. Compared with C_1_, coleoptile lengths in C_2_ and C_3_ decreased by 19.21 and 20.83%, respectively (Figure 1 and Supplementary Table 2).

In C_1_ the coleoptile length of landraces was the greatest followed by dryland cultivars with the coleoptile lengths of the irrigated cultivars being the shortest. Among the test materials, the coleoptile of Yulanmai was the longest and that of Shengmai 104 was the shortest. In C_2_ the greatest decrease in coleoptile length occurred in landraces (28.73%) versus dryland cultivars (14.12%) or irrigated cultivars (20.78%), indicating that landraces were more sensitive to drought stress and dryland cultivars were more adapted to stress conditions (Table 1). Linfeng 3 and Chang 5222 were the most sensitive to drought stress, with a decrease in coleoptile length by 77.31 and 67.41%, respectively. In C_3_, the coleoptile length of most cultivars was reduced indicating that light had a significant effect on the coleoptile length (Table 1). In the field, the wheat coleoptile is generated in a darkness within the soil, therefore darkness must be used experimentally to simulate the natural environment. Seven cultivars, including Jinmai 47, Zeyou 2 (Jinmai 76), Changmai 6686, Jintai 9923, Jinmai 102, Chang 5804 and Chang 6154, showed no significant differences in coleoptile length under the three conditions and were thus insensitive to the treatment conditions.

**TABLE 1:** Coleoptile length of irrigated cultivars, dryland cultivars, and landraces in various environments.

	Irrigated cultivars			Dryland cultivars			Landraces		
	Range	Mean	CV%	Range	Mean	CV%	Range	Mean	CV%
E_1_	2.48–6.47	4.26 ± 0.80	18.76	3.10-6.63	4.60 ± 0.75	16.42	3.27–7.10	5.70 ± 0.70	12.26
E_2_	2.48–6.38	4.05 ± 0.81	19.96	2.84–6.52	4.24 ± 0.79	18.52	2.93–6.92	5.08 ± 0.74	14.53
E_3_	2.92–5.98	3.98 ± 0.73	18.35	2.54–6.36	4.13 ± 0.78	18.78	2.67–7.06	5.08 ± 0.81	15.85
E_4_	2.80–6.02	4.09 ± 0.68	16.70	3.08–6.05	4.32 ± 0.66	15.35	3.31–7.03	5.29 ± 0.64	12.05
E_5_	0.78–6.28	3.30 ± 1.10	33.47	0.60–8.12	3.73 ± 1.17	31.50	1.44–6.68	3.69 ± 1.52	41.10
E_6_	1.10–6.28	3.19 ± 1.07	33.57	0.70–6.90	3.69 ± 1.10	29.77	1.57–7.60	3.85 ± 1.60	41.49
E_7_	1.30–6.28	3.24 ± 1.02	31.44	1.03–6.54	3.71 ± 1.02	27.59	1.57–6.68	3.77 ± 1.52	40.39
E_8_	2.00–5.28	3.28 ± 0.49	15.00	2.28–4.64	3.32 ± 0.52	15.59	2.37–7.04	4.14 ± 0.87	21.10
E_9_	1.80–4.60	3.29 ± 0.54	16.43	1.70–4.70	3.35 ± 0.59	17.50	3.00–6.10	4.19 ± 0.75	17.97
E_10_	2.03–4.94	3.29 ± 0.50	15.23	2.00–4.62	3.33 ± 0.54	16.21	2.98–6.57	4.16 ± 0.73	17.55

### Effects of genotype, environment and their interactions

Broad-sense heritability (*H*^2^) is defined in each environment and BLUP value. The estimated *H*^2^ for the coleoptile length was greater than 85% (*H*^2^_(C1)_ = 87.62%; *H*^2^_(C2)_ = 87.17%;*H*^2^_(C3)_ = 91.84%) under the three conditions, indicating the trait was mainly influenced by heredity. *H*^2^ of BLUP value was 66.43%, indicating that phenotypic variation was affected by environmental factors. The different *H*^2^ values showed that there was G × E interaction for coleoptile length, but the effect of genotype was greater than that of environment. Both C_2_ and C_3_ produced a significant reduction in coleoptile length compared with C_1_, indicating that environmental differences caused overall phenotypic variation (Supplementary Table 2). Pearson’s coefficient of correlation in the three conditions, C_3_ was the highest, C_2_ was the second, and C_1_ was the lowest (Supplementary Table 3). The correlation coefficients of coleoptile length among environments were highly significant and ranged from 0.23 to 0.96 (*P* < 0.01), which also indicated that coleoptile length was affected by the environment.

### Growth curve analysis of coleoptile length

**FIGURE 2:**
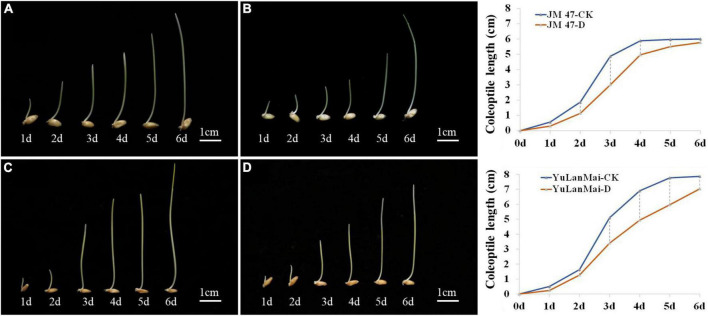
Dynamic changes of coleoptile length in a growth cycle. **(A)** Growth dynamics of Jinmai 47 under C_1_; **(B)** Growth dynamics of Jinmai 47 under C_2_; **(C)** Growth dynamics of Yulanmai wheat under C_1_; **(D)** Growth dynamics of Yulanmai under C_2_; The graph on the right is the growth curve of Jinmai 47 and Yulanmai respectively (X is the growth days, Y is the coleoptile length).

The accessions were divided into two types according to the DTC (drought tolerance coefficient) values. The coleoptile length of the insensitive cultivars such as Jinmai 47, Jinmai 919 and Linyou 2069 did not change significantly across environments. The sensitive cultivars such as Chang 6388, Shengmai 20 and Yulanmai showed clear changes in the coleoptile length across environments. Jinmai 47 has been used as a trial reference in the dryland wheat regional trial in the northern part of Huanghuai wheat region since it was released in the 1990s (Zheng et al., 2018). Yulanmai is an excellent landrace that was widely grown in the 1950s and 1960s. By investigating the dynamic changes of coleoptile length of Jinmai 47 and Yulanmai over seven days (Figure 2), the overall trend of the two cultivars was the same with the growth rate highest from day 2 to day 3, and the coleoptile length reaching a maximum from day 5 to day 6. However, the growth rate changes differed. The growth rate of Jinmai 47 under C_1_ was higher than that under C_2_ from day 1 to day 3 and lower than that under C_2_ from day 3 to day 6. Specifically, the maximum coleoptile length under stress conditions decreased by 3.99% compared with C_1_. However, the growth rate of Yulanmai under C_2_ from day 1 to day 5 was lower than that under C_1_ and the maximum coleoptile length decreased by 10.67% compared with C_1_. Thus, the differences in the growth rate of the cultivars under drought stress led to different coleoptile lengths.

### Association of coleoptile length with plant height-related traits

To determine whether the coleoptile length of the 282 wheat accessions was associated with plant height-related traits, correlation analyses was performed between coleoptile length and each of the assessed agronomic traits. The coleoptile length was significantly positively correlated (*P* < 0.05, *P* < 0.01) with plant height, internode length below the spike, and the internode length of basal sections I–IV (Supplementary Table 4). The correlation coefficients between coleoptile length and the plant height-related traits were significant in modern cultivars, but not significant in landraces (Supplementary Tables 5–7). The highest correlation coefficient was between coleoptile length and plant height. The Pearson’s coefficient of correlation between coleoptile length and the length of each stem section was higher in basal sections II and III, followed by that for the lower internode below the spike and basal section IV with the lowest correlation for basal section I (Supplementary Table 4). Except for basal sections I and IV, the correlation in the irrigated cultivars was significantly higher than in dryland cultivars. These results indicate a greater coleoptile length at the seedling stage of wheat may result in an increase of the plant height and the length of each stem section at the adult stage.

### Coleoptile length loci identified by genome-wide association study

A total of seven MTAs were detected using MLM, but only *1A_298114344* was stably associated (Supplementary Table 8). A total of 53 MTAs distributed on all chromosomes except 3D, 4B, 4D, and 7D were identified via 3VmrMLM. Among these, 13, 34, and 6 were on genomes A, B, and D, respectively, and the phenotypic variation explanation rate (*R*^2^) ranged from 2.69 to 14.23% (Supplementary Table 9). 3VmrMLM detected more stable loci and previously reported loci, possibly because all types of effects can be estimated via 3VmrMLM (Li et al., 2022). 3VmrMLM first scanned all markers at the genome-wide level to obtain potential loci, put them into the multi-locus model for selection, and obtained significant loci (Li et al., 2022). Therefore, 3VmrMLM is better at detecting stable loci compared with MLM. Five MTAs were detected by both models.

**FIGURE 3:**
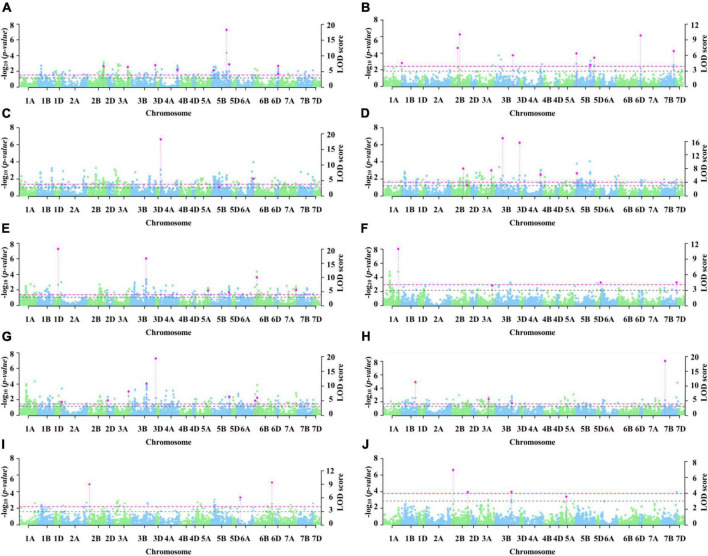
Manhattan plot of significant loci for coleoptile length. The left y axis reports -log_10_(*p*-values), which are obtained from single-marker genome-wide scanning for all the markers in the first step of 3VmrMLM, and the right y axis reports LOD scores, which are obtained from a likelihood ratio test for significant and suggested QTL in the second step of 3VmrMLM. In the Manhattan plots, the dashed pink line represents the threshold LOD = 3.99 and the dashed black line represents the threshold LOD = 3.0 (**A–J** represent E_1_, E_2_, E_3_, E_4_, E_5_, E_6_, E_7_, E_8_, E_9_, and E_10_, respectively).

In total, nine stable genetic loci were identified via 3VmrMLM under three conditions (Table 2 and Figure 3). Three loci with *R*^2^ from 3.47 to 5.72% were identified under C_1_, three loci with *R*^2^ from 4.23 to 7.79% were identified under C_2_, and two loci with *R*^2^ from 3.80 to 5.33% were identified under C_3_. *3B_810370914* with an *R*^2^ from 2.94 to 4.30% was detected in both C_1_ and C_2_. According to the Zheng et al. (2021) analysis of LD attenuation distance, the MTAs *4A_597823528* and *4A_604382166* were the same locus and explained 3.47–4.00% of the phenotypic variation. The loci significantly associated with *2B_60980523*, *3A_650566192*, *3B_519815931*, *5B_68179721*, and *5B_610798888* have not been reported previously.

**TABLE 2:** List of loci detected by genome-wide association study using the 3VmrMLM.

Marker	Chr	Position (Mb)	Env	*P*-value	*R*^2^ (%)	References
*2B_60980523*	2B	60.98	E_9_	8.14E-10	5.33	
			E_10_	1.38E-07	3.80	
*3A_650566192*	3A	650.57	E_1_	2.84E-07	3.91	
			E_4_	4.35E-08	4.49	
*3B_810370914*	3B	810.37	E_4_	6.00E-16	2.94	Ma et al. (2020)
			E_7_	1.02E-19	4.30	Ma et al. (2020)
*3B_519815931*	3B	519.82	E_5_	2.20E-17	6.51	
			E_7_	2.47E-11	4.23	
*3B_559967593*	3B	559.97	E_8_	6.57E-05	4.34	Zanke et al. (2014)
			E_10_	7.46E-05	4.28	Zanke et al. (2014)
*4A_597823528*	4A	597.82	E_4_	7.05E-07	3.47	Rebetzke et al. (2007a,2014)
*4A_604382166*	4A	604.38	E_1_	2.37E-06	4.00	Rebetzke et al. (2007a,2014)
*5B_68179721*	5B	68.18	E_1_	3.61E-06	4.21	
			E_2_	2.93E-07	5.72	
			E_4_	4.24E-07	5.62	
*5B_610798888*	5B	610.80	E_5_	1.71E-05	4.64	
			E_7_	5.52E-07	5.65	
*6B_118976998*	6B	118.98	E_5_	9.61E-11	7.79	Zanke et al. (2014), Ma et al. (2020)
			E_7_	1.33E-06	4.44	Zanke et al. (2014), Ma et al. (2020)

### Gene analysis of functional interval about major loci

To understand the biological significance among genes, these genes were annotated by Gene Ontology (GO) analysis. Overall, 514 high confidence genes from the nine functional intervals were annotated. We found that the GO domains were mainly enriched in the molecular function, including binding and catalytic activity. In the region harboring *2B_60980523*, five of these genes were involved in response to oxidative stress according to gene functional annotations in the GO public database. In the region harboring *3B_559967593*, three genes encoding expansin, including *TraesCS3B02G347800, TraesCS3B02G347900*, and *TraesCS3B02G348000*. In the region harboring *3B_810370914*, we identified three genes encoding peroxidase, including *TraesCS3B02G577900, TraesCS3B02G578000*, and *TraesCS3B02G578600*. *TraesCS4A02G298900* on chromosome 4B was involved in the regulation of seed germination. In addition, we identified seven genes that respond to defense conditions, including *TraesCS3B02G579300, TraesCS3B02G579400, TraesCS3B02G584700, TraesCS3B02G349500, TraesCS 3B02G349600, TraesCS3B02G349600, TraesCS3B02G349800. TraesCS5B02G439000* was associated with a light intensity stimulus.

### Tissue-specific expression analysis of functional interval genes

**FIGURE 4:**
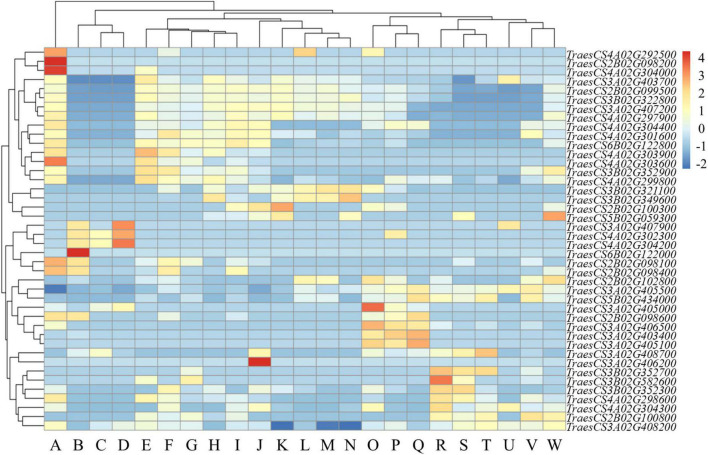
Expression profile of functional interval genes. Based on the *Z*-score of the TPM plotting Heatmap, – 2 to 4 represent expression levels from low to high. (A: coleoptile; B: grain; C: endosperm; D: aleurone layer; E: stem axis; F: first leaf sheath; G: seedling; H: first leaf blade; I: shoots; J: leaf; K: flag leaf; L: glumes; M: awns; N: flag leaf blade; O: lemma and rachis; P: spikelets; Q: stigma or ovary; R: radicle; S: roots; T: root apical meristem; U: embryo; V: shoot apical meristem; W: stem).

The expression levels of genes in functional interval in various tissues were analyzed. The results revealed high variation among the expression patterns of different tissues. Some genes in functional interval had higher expression levels in coleoptiles, and these genes had also higher expression in first leaf, stems axis and shoots. However, some genes in functional interval had relatively high expression levels in the coleoptiles, while these genes were expressed at relatively low levels in roots, endosperm, and radicle (Figure 4).

We found that *TraesCS2B02G098200* was only expressed on coleoptiles (Figure 4). *TraesCS2B02G098200* is homologous to *OsPrx116* (*Os07g49360*) in rice. The functional annotation of *TraesCS2B02G098200* was for the peroxidase activity. Peroxidase is an important antioxidant in plants, which can alleviate oxidative damage under abiotic stress (Gao et al., 2010; Qayyum et al., 2021). Therefore, the specific expression of *TraesCS2B02G098200* gene in the coleoptile could improve the tolerance of wheat to stress, thereby maintaining early vigor.

### Trend of drought tolerance coefficient values with years and frequency of favored alleles at associated loci

**FIGURE 5:**
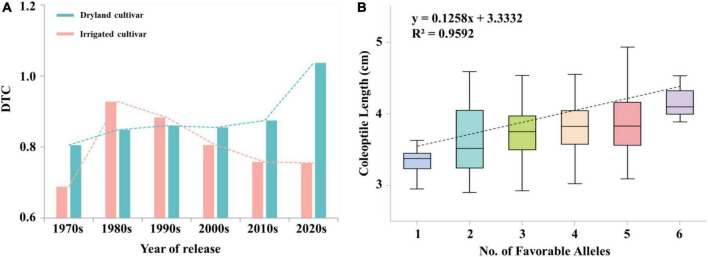
**(A)** The trend of DTC values for irrigated cultivars and dryland cultivars over time. **(B)** Linear regression between the number of favorable alleles in the population and coleoptile length. X and Y in the equation represent the number of favorable alleles and the coleoptile length, respectively.

There was a significant difference in the trend of DTC values between irrigated cultivars and dryland cultivars with year of release. DTC values were higher in dryland cultivars (0.83) than in irrigated cultivars (0.79). Figure 5A, indicating the effect of artificial selection by dryland cultivars was higher than in modern cultivars. The overall DTC value trend over time for dryland cultivars was stable at 0.85 before the 2010s. The DTC value of dryland cultivars increased to 1.04 after 2010s, indicating that breeders further increased the effect of artificial selection. The DTC values of irrigated cultivars decreased over time, which mainly due to the improvement of soil irrigation level and the decrease of attention by breeders to coleoptile length.

The frequency of favored alleles within the 282 materials was the highest in landraces (58.81%) and lowest in irrigated cultivars (40.44%). Huoshaotou had the highest frequency of favored alleles (88.89%) and exhibited a greater coleoptile length. In seven cultivars, including Jinmai 47, Zeyou 2 (Jinmai 76), Changmai 6686 and Jintai 9923, the favorable alleles had a significant positive effect at *3B_810370914*, *4A_597823528*, *5B_681790721*. In addition, the proportion of favored allele for each locus was different, which indicated that these important loci had experienced different degrees of selection during wheat breeding. For example, the frequencies of favored alleles for loci *3B_810370914* and *4A_597823528* were 93.26 and 86.17%, respectively, whereas the frequency for locus *3B_519815931* was only 8.87%.

## Discussion

### Coleoptile length is one of the main indicators for drought resistance in seedling stage

Drought is the main limiting factor for increasing yield, especially at the seedling stage of wheat (Sadok, 2017; Lin et al., 2019). In arid areas, longer wheat coleoptiles help overcome water limitation be enabling deeper planting for better stand establishment. This trait has been widely recognized by breeders (Ma et al., 2020). The coleoptile length of landraces in Shanxi Province is longer, making them more suitable for local semi-arid climate (Figure 1). Wheat with long coleoptiles emerged more frequently than wheat with short coleoptiles, especially when sown deep (Rebetzke et al., 2005, 2007a,2007b). Deeper sowing also assists in reducing removal of seeds by birds and rodents and in avoiding phytotoxicity associated with some pre-emergent herbicides (Brown et al., 2003). Selection of wheat cultivars with long coleoptile is an important component of improving emergence, weed suppression and grain yield in low rainfall regions (Farhad et al., 2014). Thus, the coleoptile length is essential for successful emergence and early plant vigor in arid and semi-arid regions. Nutrients in seeds are consumed during the growth of coleoptile. The main factor affecting coleoptile elongation is moisture (Rebetzke et al., 2007a; Sun et al., 2022). In the present study, the growth states of coleoptiles under various conditions were consistent in general, but the growth rates were quite different. Coleoptile elongation is mainly the extension of coleoptile cells. The turgor pressure distributed in the epidermis and internal tissues provides the impetus for cell elongation (Li and Wang, 1996; Chen and Chen, 2002). The expansion of coleoptiles is closely related to cell wall elongation, keeping the cell wall in a relaxed state (Gao et al., 2008). Under water stress, cell elongation is inhibited due to decreased sensitivity of cell wall (Wang et al., 1999). For example, the longitudinal growth of the coleoptiles of Jinmai 47 and Yulanmai under normal water conditions (C_1_) were maintained by continuous turgor pressure, and the cell wall did not significantly restrict elongation. Under water limited conditions (C_2_), the elongation growth of Jinmai 47 and Yulanmai was restricted by the cell wall and the maximum elongation rate was significantly reduced. Yulanmai was sensitive to water deficit, whereas Jinmai 47 was not significantly affected. Compared with Jinmai 47, the growth rate of Yulanmai decreased significantly during the growth of coleoptiles, resulting in a significant shortening of coleoptile length. Thus, the coleoptile length is one of the indicators of drought resistance ability and it can be measured at the seedling stage for selection purposes.

No consistent relationship between coleoptile length and plant height has been established. For 124 European modern winter wheat cultivars, plant height and coleoptile length showed a significant positive correlation, but the correlation between coleoptile length and the length of each section in the stem was not analyzed (Liatukas and Ruzgas, 2011). There was no correlation between coleoptile length and plant height for 707 Chinese landraces in China (Ma et al., 2020). Landraces were used in the present study as well as modern cultivars, which are more easily used in breeding. Consistent with previous studies, the correlation between plant height-related traits and coleoptile length was significant in the modern cultivars but not significant in the landraces. The coleoptile length of the modern cultivars was positively correlated with the plant height- related traits (*P* < 0.05 or *P* < 0.01). Thus, the increase of the coleoptile length of the modern cultivars could possibly promote the increase of the length of each stem section at the adult stage, and eventually lead to the increase of the plant height.

### Novel loci related to coleoptile length

A total of nine stable loci were identified via 3VmrMLM in all ten datasets. The B genome may have more genes controlling coleoptile length (Sidhu et al., 2020). In the present study, 58.14% of the MTAs were located on the B genome and only 27.91% were located on the A genome, which supports the important role of the B genome for the coleoptile length trait. Three loci identified in the present study, *3B_810370914*, *4A_597823528*, and *6B_118976998*, likely have been reported previously. Rebetzke et al. (2007a,2014) reported the DArT marker gwm637 and SNP marker *wsnp_Ex_c13615_21393511* for coleoptile length on chromosome 4A. These markers coincide with the physical location of *4A_597823528.* Ma et al. (2020) reported the locus *QCl. sicau-3B.2* for coleoptile length on chromosome 3B. This marker coincides with the physical location of *3B_810370914*, indicating that these two markers likely represent the same locus. Similarly, *QCI.sicau-6B.1* coincided with the physical location of *6B_118976998*, which indicated that two markers likely represent the same locus. In addition, *3B_559967593* and *6B_118976998* were reported to be significantly associated with plant height (Zanke et al., 2014). In the present study, *3B_559967593* was also identified as associated with coleoptile length and was located 6.31 Mb distant from the marker *BobWhite_c19725_1329* of plant height on chromosome 3B (Zanke et al., 2014). Similarly, we also identified *6B_118976998*, which was approximately 3.03Mb distant from the marker *BS00084314_51* of plant height on chromosome 6B (Zanke et al., 2014). The results indicate that *3B_559967593* and *6B_118976998* are pleiotropic, affecting both plant height and coleoptile length (Zanke et al., 2014). The loci associated with coleoptile length located on 2B (60.98 Mb), 3A (650.57 Mb), 3B (519.82 Mb), and 5B (68.18 Mb and 610.80 Mb) have not been reported previously and are likely novel. These results not only show the accuracy of the association analysis in the present work, but also confirm the high efficiency of 3VmrMLM for QTL mapping. The phenotypic variation explanation rate of these loci ranged from 2.94 to 7.79%, which is consistent with results from a previous study (Merrick et al., 2021). Among the markers identified, *6B_118976998* had the highest effect, which could explain up to 7.79% of the phenotypic variation, followed by *3B_519815931*, which explained 6.51%. There is a significant correlation between plant height and coleoptile length in wheat with dwarfing genes compared to wheat without dwarfing genes (Worland and Sayers, 1995; Rebetzke et al., 2007a). Five MTAs were detected by both 3VmrMLM and MLM. Among these MTAs, we identified *7B_702845873*, which was about 8.39 Mb distant from the *Rht13* gene (Ellis et al., 2005). Li et al. (2015) and Tian et al. (2017) reported the markers *Xbarc103* and *Xwmc256* for the dwarf gene *Rht24* (*QPH.caas-6A*) on chromosome 6A. These markers coincided with the physical location of *6A_585345446* in the present study, indicating that all three markers are linked to the *Rht24* locus. These results indicated that the dwarfing genes *Rht13* and *Rht24* are associated with reductions in both plant height and coleoptile length. Therefore, the positive correlation between coleoptile length and plant height parameters among Chinese modern cultivars and its absence in Chinese landraces could be due to the Green Revolution in China in the 1960s, which was the result of breeding (Peng, 1999; Li and Shan, 2022).

### Molecular markers of coleoptile length are feasible in breeding

Breeders seek to maintain long coleoptile lengths in semi-dwarf genotypes (Li et al., 2017). However, it is a challenge to breed semi-dwarf long-coleoptile wheat cultivars, which due to the complex relationship between coleoptile lengths and plant height. Landraces have wide adaptability to local environments, which had the highest numbers of favorable alleles. The number of favored alleles in dryland cultivars was slightly higher than that in irrigated cultivars among the modern cultivars, which was consistent with the shorter coleoptile length and lower favorable allelic variation frequency for the trait in irrigated cultivars. This result is likely because dryland cultivars are dependent on rainfall, whereas irrigated cultivars are dependent on soil irrigation levels. Since the advent of the Green Revolution, the negative selection of coleoptile favorable alleles has been excessive during positive selection for reduced plant height (Spielmeyer et al., 2007). Therefore, the breeding of long coleoptiles in wheat should focus on dryland cultivars and landraces in the future, which could enable combining longer coleoptiles with semi-dwarf plant height. Among seven cultivars that were insensitive to the treatment conditions in the present study the number of favorable alleles in Jinmai 47, Zeyou 2 (Jinmai 76), Changmai 6686, and Chang6154 was higher than in other three cultivars, and loci *3B_810370914*, *4A_597823528*, and *5B_68179721* all had favorable alleles. This result may explain the high seedling emergence rate and stress resistance of Jinmai 47, which has been used as a trial reference in the dryland wheat regional trials. Six loci had additive effects toward increasing coleoptile length, including *3A_650566192*, *3B_559967593*, *3B_810370914*, *4A_597823528*, *5B_68179721*, and *5B_610798888*. The average coleoptile length increased as the number of favored alleles increased (Figure 5B). Among the six loci, *3A_650566192*, *5B_68179721*, and *5B_610798888* are novel loci related to coleoptile length and these markers can be used to develop molecular markers for selecting long coleoptile cultivars.

## Data availability statement

The raw data supporting the conclusions of this article will be made available by the authors, without undue reservation.

## Author contributions

NW and SZ performed the phenotypic evaluation, conducted data analysis, and drafted the manuscript. YL, JiW, BW, JiZ, and LQ performed the phenotypice valuation and helped with data analysis. JiZ, LQ, XZ, and JuZ helped to draft the manuscript. JuZ and JuW designed and coordinated the study and revised the manuscript. All authors have read and approved the final manuscript for publication.

## Footnotes

1
http://wheatomics.sdau.edu.cn/

2
http://www.wheat-expression.com/

3
https://www.sas.com/

4
http://www.jmp.com/

5
https://www.ibm.com

6
https://www.rstudio.com/

7
https://www.origin.com/hkg/zh-tw/store
