# Supplementary material for: Genome-wide association study of coleoptile length with Shanxi wheat
Source: Front Plant Sci. 2022 Sep 21;13:1016551. doi: 10.3389/fpls.2022.1016551 (PMC9532578; doi:10.3389/fpls.2022.1016551)
Supplement: Supplementary file 2 [file Data_Sheet_1.docx]

Supplementary Material

## Supplementary Tables

## Supplementary Table 1. Coleoptile length values of 282 Shanxi wheat accessions in all ten environments

| **Number** | **Cultivar (lines)** | **Cultivated**  **ecotype** | **Year** |
| --- | --- | --- | --- |
| 1 | Jinmai 1 | Dryland | 1973 |
| 2 | Jinmai 5 | Dryland | 1973 |
| 3 | Jinmai 11 | Irrigated | 1980 |
| 4 | Jinmai 12 | Irrigated | 1980 |
| 5 | Jinmai 16 | Dryland | 1982 |
| 6 | Jinmai 17 | Dryland | 1982 |
| 7 | Jinmai 18 | Irrigated | 1983 |
| 8 | Jinmai 19 | Irrigated | 1983 |
| 9 | Jinmai 20 | Irrigated | 1984 |
| 10 | Jinmai 21 | Dryland | 1985 |
| 11 | Jinmai 22 | Dryland | 1985 |
| 12 | Jinmai 23 | Dryland | 1985 |
| 13 | Jinmai 24 | Dryland | 1987 |
| 14 | Jinmai 25 | Irrigated | 1988 |
| 15 | Jinmai 27 | Dryland | 1989 |
| 16 | Jinmai 28 | Dryland | 1989 |
| 17 | Jinmai 29 | Dryland | 1989 |
| 18 | Jinmai 31 | Dryland | 1990 |
| 19 | Jinmai 32 | Irrigated | 1990 |
| 20 | Jinmai 30 | Irrigated | 1990 |
| 21 | Jinmai 33 | Dryland | 1990 |
| 22 | Jinmai 35 | Irrigated | 1990 |
| 23 | Jinmai 36 | Dryland | 1991 |
| 24 | Jinmai 37 | Dryland | 1991 |
| 25 | Jinmai 38 | Dryland | 1991 |
| 26 | Jinmai 39 | Dryland | 1991 |
| 27 | Jinmai 40 | Dryland | 1991 |
| 28 | Jinmai 41 | Irrigated | 1992 |
| 29 | Jinmai 42 | Dryland | 1992 |
| 30 | Jinmai 44 | Dryland | 1992 |
| 31 | Jinmai 43 | Dryland | 1992 |
| 32 | Jinmai 45 | Irrigated | 1993 |
| 33 | Jinmai 46 | Dryland | 1994 |
| 34 | Jinmai 47 | Dryland | 1995 |
| 35 | Jinmai 48 | Irrigated | 1995 |
| 36 | Jinmai 49 | Irrigated | 1996 |
| 37 | Jinmai 50 | Dryland | 1996 |
| 38 | Jinmai 51 | Dryland | 1996 |
| 39 | Jinmai 52 | Irrigated | 1996 |
| 40 | Jinmai 53 | Dryland | 1996 |
| 41 | Jinmai 54 | Dryland | 1997 |
| 42 | Jinmai 56 | Irrigated | 1998 |
| 43 | Jinmai 57 | Irrigated | 1998 |
| 44 | Jinmai 58 | Irrigated | 1998 |
| 45 | Jinmai 59 | Dryland | 1998 |
| 46 | Jinmai 60 | Dryland | 1999 |
| 47 | Jinmai 61 | Irrigated | 1999 |
| 48 | Jinmai 62 | Irrigated | 1999 |
| 49 | Jinmai 63 | Dryland | 1999 |
| 50 | Jinmai 65 | Irrigated | 2000 |
| 51 | Jinmai 66 | Irrigated | 2000 |
| 52 | Jinmai 67 | Irrigated | 2000 |
| 53 | Jinmai 68 | Dryland | 2000 |
| 54 | Jinmai 70 | Dryland | 2001 |
| 55 | Jinmai 71 | Irrigated | 2001 |
| 56 | Jinmai 72 | Irrigated | 2002 |
| 57 | Jinmai 73 | Dryland | 2002 |
| 58 | Jinmai 74 | Irrigated | 2002 |
| 59 | Jinmai 75 | Irrigated | 2002 |
| 60 | Linfen 615 | Dryland | 2002 |
| 61 | Yunyin 1 | Irrigated | 2002 |
| 62 | Jinnong 207 | Irrigated | 2002 |
| 63 | Chang 6878 | Dryland | 2002 |
| 64 | Jintai 170 | Dryland | 2002 |
| 65 | Linyou 145 | Irrigated | 2003 |
| 66 | Linfen 138 | Irrigated | 2003 |
| 67 | Chang 6154 | Dryland | 2003 |
| 68 | Hedong TX-006 | Dryland | 2003 |
| 69 | Linfeng 3 | Dryland | 2004 |
| 70 | Jintai 65 | Irrigated | 2003 |
| 71 | Linyuan 3158 | Irrigated | 2004 |
| 72 | Linkang11 | Dryland | 2004 |
| 73 | Zeyou 2 | Dryland | 2004 |
| 74 | Donghei 1 | Irrigated | 2004 |
| 75 | Yunhei 28 | Irrigated | 2004 |
| 76 | Donghei 10 | Dryland | 2004 |
| 77 | Linyou 2018 | Irrigated | 2005 |
| 78 | Linyou 2069 | Irrigated | 2005 |
| 79 | Jinchun 3 | Dryland | 1974 |
| 80 | Jinchun 13 | Irrigated | 1996 |
| 81 | Jinchun 15 | Irrigated | 2004 |
| 82 | Yunhan 2335 | Dryland | 2005 |
| 83 | Chang 6359 | Dryland | 2005 |
| 84 | Changmai 5079 | Irrigated | 2005 |
| 85 | Chang 6452 | Irrigated | 2005 |
| 86 | Chang 4640 | Dryland | 2005 |
| 87 | Fenheimai 1831 | Irrigated | 2005 |
| 88 | Jinmai 78 | Dryland | 2006 |
| 89 | Jinmai 79 | Dryland | 2006 |
| 90 | Jinmai 80 | Dryland | 2006 |
| 91 | Jinmai 81 | Irrigated | 2006 |
| 92 | Fen 4846 | Irrigated | 2006 |
| 93 | Fen 4439 | Irrigated | 2006 |
| 94 | Jinmai 82 | Irrigated | 2007 |
| 95 | Linfen 8050 | Irrigated | 2007 |
| 96 | Jinmai 83 | Irrigated | 2007 |
| 97 | Linfen 6510 | Irrigated | 2007 |
| 98 | Yunhan20410 | Dryland | 2007 |
| 99 | Changmai6686 | Irrigated | 2007 |
| 100 | Chang 7016 | Dryland | 2007 |
| 101 | Jinmai 84 | Irrigated | 2008 |
| 102 | Jinmai 85 | Dryland | 2008 |
| 103 | Jintai 9923 | Irrigated | 2008 |
| 104 | Jinmai 86 | Irrigated | 2008 |
| 105 | Tai 5902 | Irrigated | 2008 |
| 106 | Changmai6135 | Irrigated | 2008 |
| 107 | Jinmai 87 | Dryland | 2009 |
| 108 | Yunhan 719 | Dryland | 2009 |
| 109 | Jinmai 88 | Dryland | 2009 |
| 110 | Shannong 129 | Irrigated | 2009 |
| 111 | Tai13606 | Irrigated | 2009 |
| 112 | Changmai 5973 | Irrigated | 2009 |
| 113 | Chang 5222 | Irrigated | 2009 |
| 114 | Linyuan 8 | Irrigated | 2010 |
| 115 | Changmai 251 | Irrigated | 2011 |
| 116 | Chang 6197 | Dryland | 2017 |
| 117 | Chang 8744 | Dryland | 2011 |
| 118 | Jinmai 90 | Dryland | 2011 |
| 119 | Jinmai 91 | Dryland | 2011 |
| 120 | Yunhan 805 | Dryland | 2011 |
| 121 | Jinmai 92 | Dryland | 2013 |
| 122 | Jintai 182 | Irrigated | 2013 |
| 123 | Chang 4853 | Dryland | 2013 |
| 124 | Jinmai 94 | Irrigated | 2014 |
| 125 | Jinmai 95 | Irrigated | 2014 |
| 126 | Jinmai 96 | Irrigated | 2014 |
| 127 | Jintai 102 | Irrigated | 2014 |
| 128 | Jinmai 97 | Dryland | 2014 |
| 129 | Jinmai 98 | Dryland | 2014 |
| 130 | Taichun 3473 | Irrigated | 2014 |
| 131 | Jinmai 99 | Dryland | 2015 |
| 132 | Yunhan 21-30 | Dryland | 2003 |
| 133 | Liangxing67 | Irrigated | 2016 |
| 134 | Yunhan137 | Dryland | 2016 |
| 135 | Tai 113 | Irrigated | 2016 |
| 136 | Jinzuo 80 | Irrigated | 2016 |
| 137 | Jintai 114 | Irrigated | 2016 |
| 138 | Chang 6794 | Irrigated | 2016 |
| 139 | Chang 7080 | Dryland | 2017 |
| 140 | Jintai 1310 | Dryland | 2016 |
| 141 | Chang 6990 | Dryland | 2016 |
| 142 | Zhongmai 247 | Irrigated | 2016 |
| 143 | Linhan No. 6 | Dryland | 2006 |
| 144 | Lin Y8161 | Dryland | 2017 |
| 145 | Zhonghan 110 | Dryland | 2002 |
| 146 | lvhan 1608 | Dryland | 2004 |
| 147 | Xiaoheimai 76 | Irrigated | 1997 |
| 148 | Luke 298 | Irrigated | 2018 |
| 149 | Jintai 141 | Dryland | 2017 |
| 150 | Jintai 146 | Irrigated | 2017 |
| 151 | Tai 1305 | Dryland | 2018 |
| 152 | Tai 412 | Irrigated | 2017 |
| 153 | Yun 14 guan74 | Irrigated | 2018 |
| 154 | Yaomai 16 | Dryland | 2011 |
| 155 | Shinong 086 | Irrigated | 2017 |
| 156 | Xiangmai 23 | Irrigated | 2017 |
| 157 | Linfen 6410 | Dryland | 2019 |
| 158 | Jinmai 101 | Dryland | 2018 |
| 159 | Jinmai 102 | Dryland | 2018 |
| 160 | NC206 | Irrigated | 2009 |
| 161 | Zhongmai 175 | Dryland | 2007 |
| 162 | Shunmai 1718 | Irrigated | 2011 |
| 163 | Yunhan 1512 | Dryland | 2018 |
| 164 | Yunhan139-2 | Dryland | 2017 |
| 165 | Runmai 2 | Dryland | 2016 |
| 166 | Xiangmai 8156 | Dryland | 2017 |
| 167 | Linhan 9 | Dryland | 2018 |
| 168 | Womai 323 | Dryland | 2018 |
| 169 | Jinmai 919 | Dryland | 2018 |
| 170 | Jinmai 104 | Dryland | 2017 |
| 171 | Chang 6789 | Irrigated | 2018 |
| 172 | Lin Y7287 | Irrigated | 2018 |
| 173 | Yunhan 1411-2 | Dryland | 2018 |
| 174 | Jinmai 9 | Irrigated | 1974 |
| 175 | Jinchun 14 | Irrigated | 1999 |
| 176 | Jinchun 16 | Irrigated | 2009 |
| 177 | Jinchun 17 | Irrigated | 2017 |
| 178 | Yunmai 218 | Irrigated | 2006 |
| 179 | Lin Y8012 | Irrigated | 2018 |
| 180 | Womai 608 | Irrigated | 2018 |
| 181 | Yunhei 14207 | Irrigated | 2018 |
| 182 | Yunhei 161 | Irrigated | 2018 |
| 183 | Yunnuo 32 | Dryland | 2018 |
| 184 | Dabaimai | Landrace | 1970 |
| 185 | Xiaohongpi | Landrace | 1948 |
| 186 | Niuzhijia | Landrace | 1945 |
| 187 | Honglimai | Landrace | 1947 |
| 188 | Jiangzhouhong | Landrace | 1958 |
| 189 | Jiahongmai | Landrace | 1945 |
| 190 | Niuzhijia | Landrace | 1946 |
| 191 | Siyuehong | Landrace | 1964 |
| 192 | Sanyuehong | Landrace | 1965 |
| 193 | Baimangcao | Landrace | 1966 |
| 194 | Youmangdahongjing | Landrace | 1967 |
| 195 | Zhuchengqing | Landrace | 1968 |
| 196 | Hongtumai | Landrace | 1969 |
| 197 | Baitumai | Landrace | 1970 |
| 198 | Baitumai | Landrace | 1944 |
| 199 | Hongtoumai | Landrace | 1945 |
| 200 | Yequmai | Landrace | 1946 |
| 201 | Hongpidongmai | Landrace | 1947 |
| 202 | Baishangeda | Landrace | 1958 |
| 203 | Hongyequ | Landrace | 1959 |
| 204 | Qisifeng | Landrace | 1960 |
| 205 | Hongheshang | Landrace | 1961 |
| 206 | Xianmai | Landrace | 1962 |
| 207 | Benmai | Landrace | 1963 |
| 208 | Baikehong | Landrace | 1964 |
| 209 | Hongxiaomai | Landrace | 1965 |
| 210 | Jinguoyin | Landrace | 1966 |
| 211 | Youbailan | Landrace | 1967 |
| 212 | Baixianmai | Landrace | 1968 |
| 213 | Baigengmai | Landrace | 1969 |
| 214 | Xinchengxuexingmei | Landrace | 1970 |
| 215 | Baihuomai | Landrace | 1944 |
| 216 | Baisanyuehong | Landrace | 1945 |
| 217 | Yulanmai | Landrace | 1946 |
| 218 | Zimai | Landrace | 1947 |
| 219 | Datongxiaomai | Landrace | 1948 |
| 220 | Qisuimai | Landrace | 1963 |
| 221 | Dahongmai | Landrace | 1964 |
| 222 | Huoshaotou | Landrace | 1965 |
| 223 | Dingxingzhaixiaomai | Landrace | 1966 |
| 224 | Baishanmai | Landrace | 1967 |
| 225 | Yunhan 102 | Dryland | 2014 |
| 226 | Yunhan 22-33 | Dryland | 2005 |
| 227 | Hanyou 6 | Dryland | 2020 |
| 228 | Linhan 5325 | Dryland | 2019 |
| 229 | Chang 5804 | Dryland | 2020 |
| 230 | Yunhan 1818 | Dryland | 2020 |
| 231 | Longmai 1 | Irrigated | 2019 |
| 232 | ZM148 | Dryland | 2017 |
| 233 | Yunhan 618 | Dryland | 2010 |
| 234 | Zhongyou 9507 | Irrigated | 2001 |
| 235 | Yunhan 115 | Dryland | 2011 |
| 236 | Jintai 1510 | Dryland | 2018 |
| 237 | Lumai 14 | Irrigated | 1993 |
| 238 | Taimai 101 | Irrigated | 2018 |
| 239 | Xinmai 296 | Irrigated | 2014 |
| 240 | Xin 6160 | Irrigated | 2009 |
| 241 | Changmai 3987 | Dryland | 2018 |
| 242 | Zeyou 1 | Dryland | 2002 |
| 243 | Shunmai 612 | Dryland | 2008 |
| 244 | Zhongyou 206 | Irrigated | 2008 |
| 245 | Jinfeng 3 | Irrigated | 2005 |
| 246 | Changzhi 5608 | Dryland | 2002 |
| 247 | Lunxuan 167 | Irrigated | 2014 |
| 248 | Zhongmai 349 | Irrigated | 2009 |
| 249 | Chang 6388 | Dryland | 2019 |
| 250 | Taimai 103 | Irrigated | 2019 |
| 251 | Jintai 1515 | Dryland | 2019 |
| 252 | Jinmai 106 | Irrigated | 2019 |
| 253 | Jintai 1508 | Irrigated | 2019 |
| 254 | Chang 5638 | Irrigated | 2019 |
| 255 | Jinmai 8 | Irrigated | 1973 |
| 256 | Changmai 6789 | Irrigated | 2017 |
| 257 | Yung 9805 | Irrigated | 2002 |
| 258 | Zhongmai 110 | Irrigated | 2019 |
| 259 | Linfen 139 | Dryland | 2006 |
| 260 | Yongmai 3 | Irrigated | 2008 |
| 261 | Yunmai 766 | Irrigated | 2019 |
| 262 | Linxuan 2035 | Irrigated | 2005 |
| 263 | Linyan 151 | Irrigated | 2019 |
| 264 | Yunmai 2064 | Irrigated | 2004 |
| 265 | Shengmai 20 | Irrigated | 2019 |
| 266 | Shengmai 104 | Irrigated | 2019 |
| 267 | Jinmai 107 | Dryland | 2019 |
| 268 | Pinyu 8155 | Dryland | 2019 |
| 269 | Donghei 1206 | Dryland | 2019 |
| 270 | Taizi 6336 | Irrigated | 2019 |
| 271 | Yun 85-24 | Dryland | 2018 |
| 272 | Jinmai 10 | Irrigated | 1974 |
| 273 | Jinmai 89 | Irrigated | 2011 |
| 274 | Jinmai 6 | Dryland | 1973 |
| 275 | Ziyou 5 | Irrigated | 2020 |
| 276 | Zimai 8555 | Dryland | 2020 |
| 277 | Linmai 5311 | Irrigated | 2020 |
| 278 | Tai 615 | Irrigated | 2020 |
| 279 | Changmai 3809 | Irrigated | 2020 |
| 280 | Chang 7170 | Dryland | 2020 |
| 281 | Linnong 4357 | Irrigated | 2020 |
| 282 | Womai 611 | Irrigated | 2020 |

**Supplementary Table 2. Coleoptile length index summary statistics for 282 materials under three conditions**

| **Environments** | **Mean** | **SD** | **Max** | **Min** | **CV** |
| --- | --- | --- | --- | --- | --- |
| E_1_ | 4.56 | 0.89 | 7.10 | 2.48 | 19.58% |
| E_2_ | 4.24 | 0.84 | 6.92 | 2.48 | 19.87% |
| E_3_ | 4.17 | 0.84 | 7.06 | 2.54 | 20.14% |
| E_4_ | 4.32 | 0.77 | 7.03 | 2.80 | 17.78% |
| E_5_ | 3.50 | 1.21 | 8.12 | 0.60 | 34.43% |
| E_6_ | 3.47 | 1.19 | 7.60 | 0.70 | 34.34% |
| E_7_ | 3.49 | 1.13 | 6.68 | 1.03 | 32.28% |
| E_8_ | 3.41 | 0.64 | 7.04 | 2.00 | 18.71% |
| E_9_ | 3.43 | 0.66 | 6.10 | 1.70 | 19.28% |
| E_10_ | 3.42 | 0.62 | 6.57 | 2.00 | 18.27% |

**Supplementary Figure 1.** **Histogram of frequency distribution of coleoptile length in each environment (A to K represent E_1_, E_2_, E_3_, E_4_, E_5_, E_6_, E_7_, E_8_, E_9_, E_10_, BLUP, respectively.)**

**Supplementary Table 3.** **Correlation between individual environmental data**

|  | E_1_ | E_2_ | E_3_ | E_4_ | E_5_ | E_6_ | E_7_ | E_8_ | E_9_ | E_10_ |
| --- | --- | --- | --- | --- | --- | --- | --- | --- | --- | --- |
| E_1_ | 1 |  |  |  |  |  |  |  |  |  |
| E_2_ | 0.660** | 1 |  |  |  |  |  |  |  |  |
| E_3_ | 0.730** | 0.720** | 1 |  |  |  |  |  |  |  |
| E_4_ | 0.894** | 0.883** | 0.910** | 1 |  |  |  |  |  |  |
| E_5_ | 0.604** | 0.528** | 0.615** | 0.651** | 1 |  |  |  |  |  |
| E_6_ | 0.546** | 0.474** | 0.566** | 0.591** | 0.850** | 1 |  |  |  |  |
| E_7_ | 0.597** | 0.520** | 0.613** | 0.645** | 0.960** | 0.963** | 1 |  |  |  |
| E_8_ | 0.284** | 0.259** | 0.300** | 0.314** | 0.282** | 0.234** | 0.268** | 1 |  |  |
| E_9_ | 0.345** | 0.268** | 0.330** | 0.352** | 0.283** | 0.242** | 0.273** | 0.762** | 1 |  |
| E_10_ | 0.335** | 0.281** | 0.335** | 0.355** | 0.301** | 0.253** | 0.288** | 0.939** | 0.938** | 1 |

**:significant at P＜0.01, *:significant at P＜0.05.

**Supplementary Table 4.** **Correlation between coleoptile length and plant height related traits in 282 Wheat accessions**

| **Coleoptile length** | **Plant height** | **Uppermost** | **basal section Ⅳ** | **basal section Ⅲ** | **basal section Ⅱ** | **basal section Ⅰ** |
| --- | --- | --- | --- | --- | --- | --- |
| P_1_-1 | 0.680** | 0.407** | 0.514** | 0.638** | 0.631** | 0.521** |
| P_1_-2 | 0.640** | 0.487** | 0.583** | 0.594** | 0.602** | 0.497** |
| P_1_-3 | 0.636** | 0.510** | 0.605** | 0.629** | 0.614** | 0.529** |
| P_2_-1 | 0.614** | 0.429** | 0.579** | 0.586** | 0.584** | 0.417** |
| P_2_-2 | 0.584** | 0.424** | 0.548** | 0.573** | 0.557** | 0.336** |
| P_2_-3 | 0.622** | 0.337** | 0.436** | 0.593** | 0.588** | 0.471** |

**:significant at P＜0.01, *:significant at P＜0.05.

**Supplementary Table 5.** **Correlation between coleoptile length and plant height related traits of irrigated cultivars**

| **Coleoptile length** | **Plant height** | **Uppermost** | **basal section Ⅳ** | **basal section Ⅲ** | **basal section Ⅱ** | **basal section Ⅰ** |
| --- | --- | --- | --- | --- | --- | --- |
| P_1_-1 | 0.674** | 0.472** | 0.525** | 0.591** | 0.522** | 0.397** |
| P_1_-2 | 0.647** | 0.500** | 0.641** | 0.575** | 0.549** | 0.395** |
| P_1_-3 | 0.622** | 0.511** | 0.581** | 0.566** | 0.589** | 0.442** |
| P_2_-1 | 0.599** | 0.415** | 0.537** | 0.524** | 0.525** | 0.259** |
| P_2_-2 | 0.546** | 0.418** | 0.493** | 0.536** | 0.528** | 0.238* |
| P_2_-3 | 0.580** | 0.326** | 0.359** | 0.448** | 0.469** | 0.370** |

**:significant at P＜0.01, *:significant at P＜0.05.

**Supplementary Table 6.** **Correlation between coleoptile length and plant height related traits of dryland cultivars**

| **Coleoptile length** | **Plant height** | **Uppermost** | **basal section Ⅳ** | **basal section Ⅲ** | **basal section Ⅱ** | **basal section Ⅰ** |
| --- | --- | --- | --- | --- | --- | --- |
| P_1_-1 | 0.589** | 0.160 | 0.444** | 0.581** | 0.589** | 0.387** |
| P_1_-2 | 0.513** | 0.323** | 0.588** | 0.487** | 0.503** | 0.425** |
| P_1_-3 | 0.524** | 0.286** | 0.586** | 0.537** | 0.524** | 0.402** |
| P_2_-1 | 0.451** | 0.322** | 0.503** | 0.525** | 0.473** | 0.293** |
| P_2_-2 | 0.453** | 0.349** | 0.488** | 0.458** | 0.395** | 0.182 |
| P_2_-3 | 0.477** | 0.246** | 0.448** | 0.516** | 0.506** | 0.294** |

**:significant at P＜0.01, *:significant at P＜0.05.

**Supplementary Table 7.** **Correlation between coleoptile length and plant height related traits of landraces**

| **Coleoptile length** | **Plant height** | **Uppermost** | **basal section Ⅳ** | **basal section Ⅲ** | **basal section Ⅱ** | **basal section Ⅰ** |
| --- | --- | --- | --- | --- | --- | --- |
| P_1_-1 | 0.111 | -0.052 | -0.150 | 0.051 | 0.115 | 0.155 |
| P_1_-2 | -0.088 | 0.013 | -0.207 | 0.056 | 0.141 | -0.065 |
| P_1_-3 | 0.006 | 0.136 | -0.241 | 0.118 | 0.117 | 0.214 |
| P_2_-1 | 0.226 | -0.087 | 0.097 | 0.022 | 0.137 | 0.29 |
| P_2_-2 | 0.010 | -0.337* | -0.101 | -0.065 | 0.040 | -0.208 |
| P_2_-3 | 0.171 | -0.044 | -0.262 | 0.242 | 0.177 | 0.247 |

**:significant at P＜0.01, *:significant at P＜0.05.

**Supplementary Table 8. List of significant MTAs detected by GWAS using MLM**

| **Marker** | **Env** | **Chr** | **Position (Mb)** | ***p*-value** | ***R^2^* (%)** | **Reference** |
| --- | --- | --- | --- | --- | --- | --- |
| 1A_298114344 | E_6_ | 1A | 298.11 | 1.97E-05 | 8.01 |  |
| 1A_298114344 | E_7_ | 1A | 298.11 | 4.86E-05 | 7.40 |  |
| 1A_536205036 | E_6_ | 1A | 536.21 | 2.59E-05 | 7.80 |  |
| 2A_5271009 | E_5_ | 2A | 5.27 | 6.42E-05 | 7.31 |  |
| 3B_154346249 | E_2_ | 3B | 154.35 | 8.28E-05 | 7.16 |  |
| 5B_552753628 | E_1_ | 5B | 552.75 | 6.99E-05 | 7.06 | Sidhu et al., 2020 |
| 6A_585345446 | E_3_ | 6A | 585.35 | 1.53E-05 | 8.20 | Rebetzke et al., 2014 |
| 7B_711460224 | E_8_ | 7B | 711.46 | 2.29E-05 | 7.77 |  |

Sidhu, J. S., Singh, D., Gill, H. S., Brar, N. K., Qiu, Y., Halder, J., et al. (2020). Genome-wide association study uncovers novel genomic regions associated with coleoptile length in hard winter wheat. *Frontiers in genetics*.10, 1345. doi: 10.3389/fgene.2019.01345

Rebetzke, G. J., Verbyla, A. P., Verbyla, K. L., Morell, M. K., Cavanagh, C. R. (2014). Use of a large multiparent wheat mapping population in genomic dissection of coleoptile and seedling growth. *Plant Biotechnology Journal*. 12(2), 219-230. doi: 10.1111/pbi.12130

**Supplementary Table 9. List of significant MTAs detected by GWAS using 3VmrMLM**

| **Marker** | **Env** | **Chr** | **Position (Mb)** | ***p*-value** | ***R^2^* (%)** | **Reference** |
| --- | --- | --- | --- | --- | --- | --- |
| 1A_536205036 | E_6_ | 1A | 536.21 | 1.13E-11 | 4.44 |  |
| 1B_606697180 | E_8_ | 1B | 606.70 | 5.34E-12 | 5.11 |  |
| 1B_29269714 | E_2_ | 1B | 29.27 | 2.88E-05 | 3.73 |  |
| 1D_260020985 | E_5_ | 1D | 260.02 | 2.05E-24 | 8.35 |  |
| 2A_5271009 | E_7_ | 2A | 5.27 | 3.26E-05 | 4.37 |  |
| 2B_337503680 | E_2_ | 2B | 337.50 | 1.03E-10 | 5.96 | Rebetzke et al., 2014 |
| 2B_60980523 | E_9_ | 2B | 60.98 | 8.14E-10 | 5.33 |  |
| 2B_529676760 | E_4_ | 2B | 529.68 | 1.37E-08 | 6.31 |  |
| 2B_200710374 | E_2_ | 2B | 200.71 | 3.85E-08 | 6.07 |  |
| 2B_60980523 | E_9_ | 2B | 60.98 | 1.38E-07 | 3.80 |  |
| 2B_665516977 | E_1_ | 2B | 665.52 | 1.66E-07 | 4.59 |  |
| 2B_690211230 | E_10_ | 2B | 690.21 | 7.82E-05 | 3.55 |  |
| 2D_19622909 | E_7_ | 2D | 19.62 | 8.93E-06 | 3.04 |  |
| 3A_685347022 | E_7_ | 3A | 685.35 | 1.04E-08 | 7.43 | Ma et al., 2020 |
| 3A_650566192 | E_4_ | 3A | 650.57 | 4.35E-08 | 4.49 |  |
| 3A_650566192 | E_1_ | 3A | 650.57 | 2.84E-07 | 3.91 |  |
| 3A_539750187 | E_8_ | 3A | 539.75 | 2.97E-06 | 5.34 |  |
| 3B_810370914 | E_7_ | 3B | 810.37 | 1.02E-19 | 4.30 | Ma et al., 2020 |
| 3B_519815931 | E_5_ | 3B | 519.82 | 2.20E-17 | 6.51 |  |
| 3B_270973209 | E_4_ | 3B | 270.97 | 3.18E-17 | 14.23 |  |
| 3B_810370914 | E_4_ | 3B | 810.37 | 6.00E-16 | 2.94 | Ma et al., 2020 |
| 3B_519815931 | E_7_ | 3B | 519.82 | 2.47E-11 | 4.23 |  |
| 3B_780960940 | E_1_ | 3B | 780.96 | 7.97E-08 | 4.63 | Ma et al., 2020 |
| 3B_609359925 | E_2_ | 3B | 609.36 | 9.65E-07 | 4.80 | Rebetzke et al., 2014 |
| 3B_559967593 | E_8_ | 3B | 559.97 | 6.57E-05 | 4.34 | Zanke et al.,2014 |
| 3B_559967593 | E_10_ | 3B | 559.97 | 7.46E-05 | 4.28 | Zanke et al.,2014 |
| 4A_25060050 | E_3_ | 4A | 25.06 | 3.92E-21 | 7.07 |  |
| 4A_597823528 | E_4_ | 4A | 597.82 | 7.05E-07 | 3.47 | Rebetzke et al., 2007a, 2014 |
| 4A_604382166 | E_1_ | 4A | 604.38 | 2.37E-06 | 4.00 | Rebetzke et al.,2007a, 2014 |
| 5A_572006336 | E_5_ | 5A | 572.01 | 4.97E-06 | 5.18 | Ma et al., 2020 |
| 5B_552753628 | E_1_ | 5B | 552.75 | 3.48E-19 | 10.91 | Sidhu et al., 2020 |
| 5B_604882065 | E_1_ | 5B | 604.88 | 4.33E-08 | 5.11 |  |
| 5B_68179721 | E_4_ | 5B | 68.18 | 2.93E-07 | 5.72 |  |
| 5B_68179721 | E_2_ | 5B | 68.18 | 4.24E-07 | 5.62 |  |
| 5B_610798888 | E_7_ | 5B | 610.80 | 5.52E-07 | 5.65 |  |
| 5B_665202775 | E_2_ | 5B | 665.20 | 2.83E-06 | 4.22 |  |
| 5B_68179721 | E_1_ | 5B | 68.18 | 3.61E-06 | 4.21 |  |
| 5B_610798888 | E_5_ | 5B | 610.80 | 1.71E-05 | 4.64 |  |
| 5B_581214437 | E_2_ | 5B | 581.21 | 6.66E-05 | 3.19 |  |
| 5D_562532607 | E_6_ | 5D | 562.53 | 3.36E-05 | 6.17 |  |
| 6A_102993644 | E_9_ | 6A | 102.99 | 9.34E-08 | 3.71 |  |
| 6A_585345446 | E_3_ | 6A | 585.35 | 6.38E-07 | 6.37 | Rebetzke et al., 2014 |
| 6B_118976998 | E_5_ | 6B | 118.98 | 9.61E-11 | 7.79 | Ma et al., 2020; Zanke et al., 2014 |
| 6B_610906829 | E_9_ | 6B | 610.91 | 3.23E-10 | 8.24 | Ma et al., 2020 |
| 6B_118976998 | E_7_ | 6B | 118.98 | 1.33E-06 | 4.44 | Ma et al., 2020; Zanke et al., 2014 |
| 6B_33068670 | E_7_ | 6B | 33.07 | 1.24E-05 | 4.77 |  |
| 6D_323453753 | E_2_ | 6D | 323.45 | 1.67E-10 | 5.40 |  |
| 6D_389928252 | E_1_ | 6D | 389.93 | 1.37E-07 | 4.73 |  |
| 6D_368378867 | E_1_ | 6D | 368.38 | 5.56E-05 | 2.69 |  |
| 7A_704467487 | E_5_ | 7A | 704.47 | 2.37E-06 | 5.41 |  |
| 7B_126043043 | E_8_ | 7B | 126.04 | 3.73E-19 | 5.13 |  |
| 7B_634861689 | E_2_ | 7B | 634.86 | 1.52E-07 | 4.79 |  |
| 7B_702845873 | E_6_ | 7B | 702.85 | 3.25E-05 | 4.22 | Rebetzke et al., 2014 |

Ma, J., Lin, Y., Tang, S., Duan, S., Wang, Q., Wu, F., et al. (2020). A genome-wide association study of coleoptile length in different Chinese wheat landraces. *Frontiers in plant science*. 11, 677. doi: 10.3389/fpls.2020.00677

Rebetzke, G. J., Ellis, M. H., Bonnett, D. G., Richards, R. A. (2007a). Molecular mapping of genes for coleoptile growth in bread wheat (*Triticum aestivum L.*). *Theoretical and Applied Genetics*. 114(7), 1173-1183. doi: 10.1007/s00122-007-0509-1

Rebetzke, G. J., Verbyla, A. P., Verbyla, K. L., Morell, M. K., Cavanagh, C. R. (2014). Use of a large multiparent wheat mapping population in genomic dissection of coleoptile and seedling growth. *Plant Biotechnology Journal*. 12(2), 219-230. doi: 10.1111/pbi.12130

Sidhu, J. S., Singh, D., Gill, H. S., Brar, N. K., Qiu, Y., Halder, J., et al. (2020). Genome-wide association study uncovers novel genomic regions associated with coleoptile length in hard winter wheat. *Frontiers in genetics*.10, 1345. doi: 10.3389/fgene.2019.01345

Zanke, C. D., Ling, J., Plieske, J., Kollers, S., Ebmeyer, E., Korzun, V., et al. (2014). Whole genome association mapping of plant height in winter wheat (*Triticum aestivum L.*). *PloS one*. 9(11):e113287. doi: 10.1371/journal.pone.011328
